# Music Interventions and Delirium in Adults: A Systematic Literature Review and Meta-Analysis

**DOI:** 10.3390/brainsci12050568

**Published:** 2022-04-28

**Authors:** Jelena Golubovic, Bjørn Erik Neerland, Dagfinn Aune, Felicity A. Baker

**Affiliations:** 1 Centre for Research in Music and Health, Norwegian Academy of Music, 0363 Oslo, Norway; felicity.baker@unimelb.edu.au; 2 Creative Arts and Music Therapy Research Unit, The University of Melbourne, Melbourne 3010, Australia; 3 Oslo Delirium Research Group, Department of Geriatric Medicine, Oslo University Hospital, 0462 Oslo, Norway; bjonee@ous-hf.no; 4 Department of Epidemiology and Biostatistics, School of Public Health, Imperial College London, London SW7 2AZ, UK; d.aune@imperial.ac.uk; 5 Department of Nutrition, Oslo New University College, 0456 Oslo, Norway; 6 Department of Endocrinology, Morbid Obesity and Preventive Medicine, Oslo University Hospital, 0424 Oslo, Norway; 7 Unit of Cardiovascular and Nutritional Epidemiology, Institute of Environmental Medicine, Karolinska Institutet, 171 77 Stockholm, Sweden

**Keywords:** music interventions, music therapy, delirium, acute confusion, treatment, prevention, systematic review, meta-analysis

## Abstract

Delirium is a neuropsychiatric syndrome represented by an acute disturbance in attention, awareness and cognition, highly prevalent in older, and critically ill patients, and associated with poor outcomes. This review synthesized existing evidence on the effectiveness of music interventions on delirium in adults, and music interventions (MIs), psychometric assessments and outcome measures used. We searched MEDLINE, PsychINFO, SCOPUS, Clinical Trials and CENTRAL for quantitative designs comparing any MIs to standard care or another intervention. From 1150 studies 12 met the inclusion criteria, and 6 were included in the meta-analysis. Narrative synthesis showed that most studies focused on prevention, few assessed delirium severity, with the majority of studies reporting beneficial effects. The summary relative risk for incident delirium comparing music vs. no music in postsurgical and critically ill older patients was 0.52 (95% confidential interval (CI): 0.20–1.35, I^2^ = 79.1%, heterogeneity <0.0001) for the random effects model and 0.47 (95% CI: 0.34–0.66) using the fixed effects model. Music listening interventions were more commonly applied than music therapy delivered by credentialed music therapists, and delirium assessments methods were heterogeneous, including both standardized tools and systematic observations. Better designed studies are needed addressing effectiveness of MIs in specific patient subgroups, exploring the correlations between intervention-types/dosages and delirium symptoms.

## 1. Introduction

Delirium is a complex, neuropsychiatric syndrome represented by an acutely altered mental status, and disturbed cognition, attention and arousal [1], most prevalent in acutely hospitalized older patients and in those with pre-existing dementia. Delirium also affects younger age groups, particularly critically ill patients in the intensive care units (ICUs) [2]. Delirium may precipitate dementia, or exacerbate existing cognitive impairments, and is associated with prolonged hospital stay, increased need for long term care [3,4,5] and mortality [6].

Pharmacological agents show poor effect in managing symptoms of delirium, but there is evidence in favor of supportive non-pharmacological, multifactorial approaches [7,8]. As multicomponent interventions are most effective in preventing delirium [9,10], literature highlights the need for further research on novel non-pharmacological prevention and treatment alternatives [11]. Music interventions are low-risk non-pharmacological approaches, with many known health benefits, already used in a variety of healthcare settings [12,13,14,15,16,17]. To date, a few reviews have synthesized the evidence on the effectiveness of music interventions for delirium [18,19,20,21]; however, they were not comprehensive, combined studies containing participants with other diagnosis, findings were not directly relevant for delirium and none included meta-analyses. The reviews suggest that music interventions could be effective [18] and warrant further exploration.

This study assessed the effectiveness of music interventions on prevention and/or treatment of delirium in adults (≥18 years) across clinical settings and levels of care. Our primary research question was: Are music interventions effective in preventing and treating delirium in adults? Secondary questions were: (1) What music interventions have been used with adults with delirium? (2) What standardized psychometric assessments have been used to measure the effect? and (3) What health outcomes did the music interventions aim to effect, and what were the sizes of these effects?

## 2. Materials and Methods

This review protocol follows the Preferred Reporting Items for Systematic Reviews and Meta-Analyses statement (PRISMA) [22], was submitted to PROSPERO on 10 October 2020, and registered/last edited on 3 November 2020 (ID: CRD42020212260).

### 2.1. Data Sources and Eligibility Criteria

We searched MEDLINE, PsychINFO, SCOPUS, ClinicalTrials.gov and Cochrane Central Register of Controlled Trials. Primary search terms were music and delirium in combination. Other terms commonly used to describe delirium symptoms and to describe music were also searched. We included free terms and MeSH terms, or the database’s own controlled vocabulary/thesaurus. Truncations and expanded functions were used where available (Supplementary Method S1).

No filters or limitations in the search engines of the databases were used. Search dates were for available quantitative studies from 1946 to present. The studies were uploaded to the online software Rayyan (https://rayyan.ai/cite) [23] for screening and selection and duplicates were identified and removed. Supplementary Method S2 illustrates our eligibility criteria.

### 2.2. Study Selection

Titles and abstracts were assessed for inclusion by at least two masked reviewers. Where the abstract and the title did not provide sufficient information to confirm inclusion/exclusion, the studies were included in the full text review. The decisions were made by at least two reviewers, with a third reviewer recruited to resolve disagreements. All decisions regarding the study selection and the reasons for exclusion were recorded in Rayyan software.

### 2.3. Data Extraction

One reviewer extracted the data using a tailored data extraction form which was informed by our review questions (Supplementary Method S3). Two reviewers independently checked the data for accuracy, and any discrepancies and disagreements were discussed and resolved between the reviewers.

### 2.4. Quality Assessment (Risk of Bias)

Each article meeting the inclusion criteria was subjected to a quality appraisal using the 11-item PEDro scale [24,25]. Points were awarded for items 2–11 if the criteria were clearly and undoubtedly satisfied, and no points were awarded to item 1 (Supplementary Table S1).

### 2.5. Data Analysis

#### 2.5.1. Narrative Synthesis

Heterogeneity was observed in study designs, settings, interventions and outcome measures. A narrative synthesis was undertaken using the adapted Economic and Social Research Council (ESRC) Methods Program [26]. Only steps 2 and 3 of its four-pronged framework were undertaken iteratively. Step 2, a preliminary synthesis, included an initial description of the findings as well as identifying, listing, tabulating and counting the results. Exploring the relationships within and between the studies (step 3) helped identify factors that can explain the impact of the interventions, differences in effect sizes and direction of the effects across the studies, relationship between the methodology and the findings within the studies and the variability of findings between different studies [26].

#### 2.5.2. Meta-Analysis and Statistical Methods

For homogenous studies, we performed a meta-analysis. Due to the few available studies and small sample sizes, we calculated the estimated effect of music exposure (of any kind), compared to no-exposure, on delirium incidence/prevention. No other meta-analyses were considered given the high heterogeneity for all other outcomes.

Since evidence of heterogeneity between the studies was detected, we used the random effects model to calculate summary relative risks (RR) and 95% confidence intervals (CI) [27]. The fixed effects model was also used as a sensitivity analysis to see whether the two models showed consistent results. Heterogeneity between the studies was evaluated using Q and I^2^ statistics [28], and publication bias was assessed using Egger’s test [29], as well as by inspection of the funnel plot. To assess the robustness of the summary estimate, a sensitivity analysis was conducted by excluding one study at a time and assessing its impact on the summary estimates. The statistical analysis was conducted using the Stata software (version 13.1) [30].

## 3. Results

### 3.1. Study Selection

**Figure 1:**
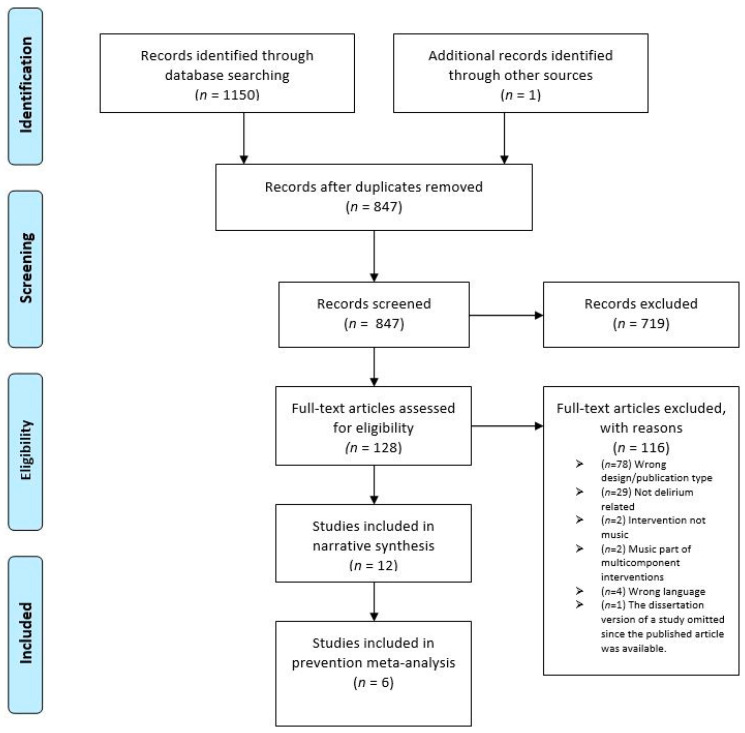
PRISMA flow diagram.

Searches performed on the 16 October 2020, and updated on the 5 October 2021, yielded a total of 1150 studies. One additional study was identified during manual reference checking and citation tracking. After the duplicates were removed, 847 studies remained and after the first screening of the titles and abstracts, 128 studies were selected for the full-text review. After the full text review by 2 reviewers, a further 14 studies required a third reviewer. Our final selection consisted of 12 studies [31,32,33,34,35,36,37,38,39,40,41,42], with the publication years ranging from 2004 to 2020, and six of the studies were included in the meta-analysis [31,33,34,36,38,42] (Figure 1).

### 3.2. Study Characteristics

#### 3.2.1. Research Designs

Two studies in our selection had a within-subject design [40,41], whereas 10 involved between-group comparisons. Seven studies were randomized controlled trials (RCTs), one an observational, prospective cohort study [42] and two non-randomized studies comparing an experimental group with a historical control group [38,39]. Five RCTs had a two-arm design involving one experimental condition [32,34,35,36,37] and two were three-armed trials comparing two experimental interventions with a control group [31,33]. All the included trials were feasibility studies (Table 1).

#### 3.2.2. Samples

The majority of the participants were mechanically ventilated patients from the postsurgical ICU units (*n* = 249) [31,33,34,42], and recovery-room patients from the surgical units (*n* = 323) [35,36,37,38]. Others were sampled from acute care units (*n* = 34) [40,41] and long-term care facilities (*n* = 78) [32,39]. The mean age of the participants across the included studies was 75.7 years. Only two trials reported a lower mean age (57.4 and 67.5) [31,42]. Eight studies included patients at risk of developing delirium, two studies involved patients with dementia/probable dementia with possible delirium as one of the symptoms of advancing dementia and two studies included patients with a delirium diagnosis at enrolment (Table 1).

#### 3.2.3. Interventions

Nine studies involved music listening (ML) interventions, two studies included music therapy (MT) interventions delivered by credentialed music therapists [32,40] and one included MT and ML [33] (Table 2).

##### Music Listening

The ML is a receptive intervention, and was usually provided by the investigators, hospital carers, family members or patients themselves. ML consisted of pre-recorded music, delivered through various musical devices (e.g., MP3 player) using loud speakers or headsets. Music was played automatically, at pre-determined hours, or at patients’ request, at any time of the day except overnight. ML protocols detailing the duration and the frequency of the music delivery were mostly not standardized and varied within and between the participants, with the reported duration of listening sessions ranging from 15–20 min to one hour, and the number of listening sessions per day varying between one and four. The total duration of the exposure to music varied widely—from 2 to 3 days, 1 to 3 weeks, and 24 weeks. The exact number of music sessions and total duration of music exposure were not always clearly reported.

ML involved either personalized, preferred music, or researcher-selected non-personalized music chosen because of its objective characteristics and known health benefits. Two studies reported using slow-tempo relaxing music (60–80 bpm) because of its simple repetitive rhythms and sedative-sparing and anxiolytic effects [31,34]. One study included baroque music because of its rhythmic nature and absence of sharp transitions in volume, which were viewed as calming and appropriate for the busy acute care hospital environment [41]. Some studies reported including lullaby music for its “soothing” properties [35,36], or classical music for being “relaxing” [42], whereas others included a broad musical selection including classical, popular, meditation music, musicals and jazz to appeal to patients’ preferences [37]. Musical preferences were assessed on admission in only three studies [31,38,39] (Table 2).

##### Music Therapy

Three studies in our selection included music therapy interventions (MTI) delivered by the credentialed music therapists [32,33,40]. MTIs consisted of shared musical interactions where the patients actively participated in the music-making process. Giovagnoli et al. [32] included a non-verbal MTI based on the free sound–music interactions and the use of rhythmical and melodic instruments. Cheong et al. [40] MTI comprised a patient-centered, improvisational approach, involving playing and improvising on familiar, patient-selected music. Kim et al. [33] incorporated music listening into the individual MTI and delivered interactive MTI during the day and personalized music listening, following a music therapist’s assessment of preferences, at night.

#### 3.2.4. Comparators

Music interventions were compared either to usual care or to another intervention (non-pharmacological or pharmacological). Where two ML interventions were compared, one was usually based on personalized and the other on non-personalized music [31,39]. One study compared listening to two different musical genres [39], and one compared ML to MTI [33] (Table 2 and Supplementary Table S2).

#### 3.2.5. Outcomes, Tools and Procedures

The incidence of delirium was mostly formulated as a binary, “yes/no” variable, and studies mainly focused on the preventive potential of music interventions. Delirium was either diagnosed by the use of standardized delirium diagnostic tools (e.g., Confusion Assessment Method for the Intensive Care Unit—CAM-ICU; Neelon, Champagne, Carlson and Funk, acute con-fusion scale—NEECHAM), or identified by reading the medical records. None of the studies described delirium subtypes.

Changes in delirium severity, and treatment effects of music interventions, were less commonly reported. Severity was assessed either directly, utilizing existing delirium-severity tools (e.g., Richmond Agitation and Sedation Scale-RASS, CAM-ICU-7), or indirectly by observing changes in other outcomes, such as physiological variables, mobility, changes in engagement, mood and emotional state, pain, anxiety, episodes of disruptive behaviors and cognitive changes, changes in sleep quality and the duration of hospital stay. In two studies [32,39], delirium was considered a symptom of advancing dementia and assessed using the Neuropsychiatric Inventory Questionnaire (NPI-Q) (Table 3).

Only two of the included studies had delirium diagnosis as the enrolment criteria [40,41], although all the studies assessed delirium pre-intervention. Delirium was usually assessed daily or several times per day, within a specific timeframe, for as long as the intervention was administered (from 2–3 days to 24 weeks). The majority of studies focused on assessing effects immediately after the interventions, and only two looked at the changes in delirium symptoms over time for sustained effects [31,32] (Table 3 and Supplementary Table S3).

### 3.3. Risk of Bias

The calculated Cohen’s Kappa coefficient (*k* = 0.75) indicated a substantial level of agreement between the two principal reviewers [43]. The PEDro scores of the included studies ranged from “excellent” (*n* = 1), “good” (*n* = 4), “fair” (*n* = 3) to “poor” (*n* = 4) (mean 4.9 ± 2.5; median 4.5). The risk of bias was usually related to the absence of participant, intervention-administrators, and assessor masking, as well as the absence of allocation concealment and randomization (Supplementary Table S1).

### 3.4. Synthesis of Results

#### 3.4.1. Direct Outcomes

Nine studies compared music-interventions (MIs) to usual care, and three compared music to another intervention. Five studies focused on prevention [33,34,36,37,38], three focused on prevention and treatment [31,35,42] and four examined treatment only [32,39,40,41]. Heterogeneity was present in study design, type of MIs and comparators, as well as assessment measures of delirium incidence and severity.

##### Music—No Music (Prevention)

Four RCTs examined delirium incidence in postsurgical orthopedic patients by comparing ML to the usual care. Two RCTs [36,37] assessed the number of delirium episodes using systematic observations and reported significant differences between the intervention and control groups (F = 29.56, *p* = 0.001; F = 19.56, *p* = 0.001). The methodological quality of these studies was “fair” [36] and “poor” [37] (Supplementary Table S1). While Johnson et al. [34] reported no delirium episodes in the two groups, McCaffrey [35] reported lower incidence of ICU delirium in the experimental group, on all 3 data-collecting days (df = 1.22, F = 7.28, *p* = 0.014). The methodological quality of these trials was assessed as “good” [35] and “fair” [34].

The prospective cohort study by Browning et al. [42] reported less proportion of time with ICU delirium in the ML groups (33%), compared to the usual care groups (67%). The non-randomized trial by Sharda et al. [38] assessed delirium in postsurgical patients using ICD codes and found lower incident delirium in the ML group (17.8% of the participants) compared to the usual care (28.7%). The outcomes of the two trials were not statistically significant, had small samples, and “poor” to “fair” methodological quality.

##### Music—No Music (Prevention Meta-Analysis)

**Figure 2:**
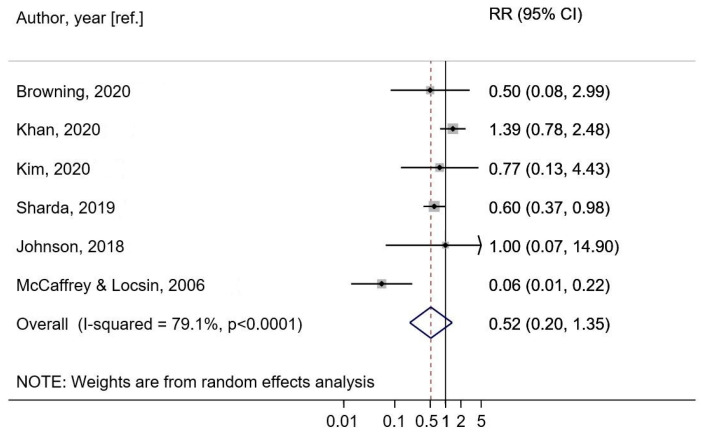
Music exposure and delirium incidence (random effects meta-analysis) [31,33,34,36,38,42].

Six studies were included in the meta-analysis of music vs. no music and delirium incidence. The summary RR for incident delirium was 0.52 (95% CI: 0.20–1.35, I^2^ = 79.1%, heterogeneity <0.0001) for the random effects model (Figure 2). The studies showed some variation in interventions and comparators, with four of them comparing ML to usual care and two including interactive MT and another intervention as a comparator. There were also variations in the musical content of the interventions, and type of participants. When studies reported results for multiple MI groups vs. a control group [31,33] we combined the results for the two intervention groups and used the combined result in the analysis for consistency with the remaining studies, which only had one intervention group [34,36,38,42].

The summary RR ranged from 0.38 (95% CI: 0.13–1.08) when excluding the study by Khan et al. [31] to 0.84 (95% CI: 0.53–1.34) (Supplementary Figure S3). In a sensitivity analysis using a fixed effects model the summary RR was 0.47 (95% CI: 0.34–0.66) (Supplementary Figure S1). Methodological qualities ranged from “poor” to “excellent” (PEDro score median 5.5; mean 5.5; SD 2.42), with the risk of bias usually related to the lacking allocation concealment and masking. There was no indication of publication bias with Egger’s test (*p* = 0.51) or by inspection of the funnel plot (Supplementary Figure S2).

##### Music—Another Intervention (Treatment)

Three treatment studies reported changes in delirium symptoms post-intervention in mechanically ventilated ICU patients [31], and LTC patients with dementia/probable dementia [32,39]. The “excellent” methodological quality RCT of Khan et al. [31] compared two MIs and one attention-control intervention and assessed delirium severity using RASS and CAM-ICU-7. Although not statistically significant, their results showed a trend towards improvement in delirium symptoms and suggested that researcher-selected slow tempo music is more effective than personalized music.

Two studies compared two ML interventions [39], or a MT intervention with a pharmacological agent [32] and assessed changes in delirium, using NPI-Q, in patients with advanced dementia. Giovagnoli et al. [32] reported no significant changes in delirium symptoms between the groups, but also no worsening of overall cognitive performance. Conversely, Correa et al. [39] found decreases in delirium symptom severity post-intervention in the group receiving personalized, popular music (t = 2.3; *p* = 0.02).

##### Music—No Music (Treatment)

Browning et al. [42] trial of a “fair” methodological quality and with a small sample, reported mean RASS score for delirium severity in mechanically ventilated ICU patients, suggesting that ML group spent more time alert and calm to agitated (1.3 ± 1.2(5)), while the control group fluctuated between sedated and agitated.

Cheong et al. [40] examined the effectiveness of MT, and Helmes and Wiancko [41] of ML in treatment of delirium in acute geriatric hospital patients. Neither of these studies reported assessment of delirium severity, nor the use of any standardized instruments. Despite their high risk of bias, these studies reported some significant changes in outcomes indirectly relevant for delirium severity (e.g., mood, engagement, and frequency of disruptive behaviors).

#### 3.4.2. Indirect Outcomes

##### Physiological Measures

Physiological variables can be biomarkers signaling physiological stress associated with the presence of delirium, and changes in these variables might thus be indicative of changes in delirium severity. Khan et al. [31] reported a significant increase in HR (*p* = 0.02) and DBP (*p* = 0.02) in the ML group, receiving researcher-selected slow tempo music compared to the personalized music group. However, Johnson et al. [34] showed that the ML group had a decrease in HR post-intervention (*p* ≤ 0.01), as well as an increase in SBP post-intervention comparing to pre-intervention (*p* ≤ 0.01), for the postoperative orthopedic ICU patients. This study also showed significant differences in SBP between the ML group and the control group.

##### Anxiety, Mood, and Engagement

Khan et al. [31] detected non-significant changes in anxiety between the groups in critically ill patients. Cheong et al. [40] reported statistically significant pre-post intervention changes in engagement and mood in patients with delirium. Notably, there was a higher frequency of positive Menorah Park Engagement Scale (MPES)—constructive and passive engagement (*p* = 0.01), and positive Observed Emotion Rating Scale (OERS)—pleasure and general alertness (*p* = 0.01), as well as lower frequency of negative MPES—self-engagement and non-engagement (*p* = 0.02), and negative OERS—anger, anxiety and sadness (*p* = 0.045). Correa et al. [39] reported more expressions of joy (*p* = 0.039) and surprise (*p* = 0.041) in the group receiving personalized, popular music compared to the non-personalized, classical music groups.

##### Sleep

Kim et al.’s [33] “excellent” quality study, reported that music was effective in promoting sleep in the critically ill patients, and thus may also prevent delirium. Results suggested that patient-directed interactive MT intervention might be more effective than ML (*p* < 0.01).

## 4. Discussion

Our meta-analysis indicated an approximately 50% reduction in risk of delirium after exposure to music compared to non-exposure in postsurgical and critically ill ICU patients. Although the results were statistically significant only in the secondary, sensitivity analysis using a fixed effects model, and not in the primary random effects analysis, the summary estimate was similar for the two models. Our narrative synthesis showed that most studies reported some beneficial effects of MIs on direct or indirect delirium outcomes, although the results were not always statistically significant. The majority of the studies involved receptive, ML interventions, while few examined the effects of expressive, improvisational MT.

Due to the few available homogenous studies, we were not able to make strong claims as to which type of MIs are the most effective for specific delirium symptoms. However, there are indications that ML might be more effective than usual care, pharmacological treatment, and other attention-control interventions in management of delirium. More studies with larger sample sizes are, therefore, necessary to confirm these hypotheses.

There is strong evidence on the correlation between anxiety [44], sleep disturbances [45] and delirium incidence [44]. Furthermore, changes in engagement and mood might be considered indicators of the improvement in delirium severity [46]. Improvisational MT showed promising effects on improving engagement, mood, anxiety, depression symptoms and sleep quality in three studies from our selection. The reported effects could indicate the potential role of MT interventions in treatment of these delirium symptoms, and in facilitating otherwise regular treatment (e.g., medication, procedural support, physiotherapy, etc.). More evidence is needed to substantiate these claims.

Compared to other studies involving pharmacological and non-pharmacological agents, studies on MIs showed heterogeneity concerning delirium outcomes and assessments, as several different diagnostic tools and procedures were used. Due to the complexity of delirium, and the multifaceted nature of MIs, it might be necessary to combine different direct and indirect measures in future research.

Delivery and dosage of MIs were not standardized in the majority of studies, which might influence the reliability of our claims. This can be attributed to the complex nature of the MIs themselves, the fluctuating nature of delirium, the challenges concerning the availability of a researcher to provide the intervention at the exact time needed, as well as the culture of acute medicine and the busy hospital environment. Music preferences were not always systematically assessed, despite the majority of studies emphasizing the importance of patients’ involvement in choosing the music.

Most studies in this review reported a high adherence in the music groups, and cost-efficient interventions. Patient-survey data revealed high participant enjoyment of the MIs [31], which might also serve as additional argument for further exploration of their utility in management of delirium.

### Strengths and Limitations

While our review asked focused questions, and we implemented a sensitive and comprehensive search strategy, our broad inclusion criteria led to high heterogeneity of participant samples and therefore limited the generalizability of the findings. Some relevant data may have been omitted due to the exclusion of the studies where music was applied as a part of the multicomponent interventions. Nevertheless, including such studies would have made it difficult to isolate the specific effects of music on delirium from the effects of other components.

This study included a narrative synthesis and meta-analysis. The narrative synthesis highlighted the possibility for applying statistical methods, and the results of our meta-analysis allowed for more specific claims about the effectiveness of music interventions on prevention of postoperative delirium in older patients. We could not make claims related to whether ML is more efficient in prevention than MT, nor which type of MIs were more efficient for prevention and which for treatment. As none of the studies involved systematic subtyping of delirium or standardized interventions, we could also not make any conclusions as to which interventions related better to which subtypes or symptoms, nor which dosage/delivery was optimal.

Only six studies were included in our meta-analysis, with allocation concealment and masking lacking in the majority of them, and with one study also lacking randomization; thus, indicating relatively high risk of bias. Given that the power in a meta-analysis depends both on the effect size, variance, heterogeneity, number of studies and sample size in the studies, our meta-analysis may be considered powered to detect a summary effect size. Conducting both the Chi-squared test and the I-squared test to detect heterogeneity and inconsistencies across the studies is a strength, given that the Chi^2^ is less powered when few studies with small samples are included, whereas the I^2^ test gives an estimate that is less dependent on the number of included studies and more focused on the impact of the heterogeneity on the meta-analysis. The I^2^ result of 79.1% shows that the variability in observed effects can be attributed to the substantial heterogeneity among the included studies, and that the result of our meta-analysis is thus not robust and should be considered as only explorative, warranting more and better designed research.

In conclusion, this review presents the evidence on MIs potentially being effective in prevention of postoperative delirium in older adults, based on the meta-analysis of the data from six clinical studies, with substantial heterogeneity, small samples and high risk of bias. More high-quality studies with larger homogenous samples are necessary to substantiate the inferences about the application and effectiveness of MIs in treatment/prevention of delirium in specific patient groups, as well as about correlations between different types and dosages of MIs, and particular delirium symptoms.

## Figures and Tables

**Table 1:** Characteristics of the included trials.

Study ^1^ and Design	Setting and Participants	Mean Age (±SD) ^2,3^	Enrolment Criteria (Delirium-Related)	Number of Participants
Khan et al., 2020 [31] RCT (3 gr.)	Medical and surgical ICU (mechanically ventilated patients)	Total: 57.4 (±14.2)	Delirium risk (not diagnosed at enrolment)	Enrolled: *n* = 56 Data analyzed: 52
Giovagnoli et al., 2018 [32] RCT (2 gr.)	LTC facilities or outpatient hospitals (moderate Alzheimer’s patients)	M-AMT: 74.3 (±5.7) M:72.0 (±7.3)	Probable dementia, (delirium symptom of advancing dementia)	Enrolled: *n* = 45 Data analyzed: 43
McCaffrey and Locsin, 2006 [36] RCT (2 gr.)	Postoperative orthopedic unit (hip/knee patients)	Total: 75.7 (±6.1) EG:76.8 (±5.1) CG:77.3 (±5.4)	Delirium risk (not diagnosed at enrolment)	Enrolled: *n* = 126 Data analyzed: 124
McCaffrey 2009 [35] RCT (2 gr.)	Postoperative orthopedic unit (hip/knee patients)	EG:74.5 (±4.8) CG:75.9 (±1.2)	Delirium risk (not diagnosed at enrolment)	Enrolled: *n* = 22 Data analyzed: 22
Kim et al., 2020 [33] RCT (3 gr.)	Postoperative ICU (postsurgical patients)	IMT:74.6 (±5.2) PML:72.3 (±4.7) CG:74.1 (±6.7)	Delirium risk (not diagnosed at enrolment)	Enrolled: 147 Data analyzed: 133
Johnson et al., 2018 [34] RCT (2 gr.)	TICU and TOU (postsurgical patients)	Total: 71.8 (±9.2)	Delirium risk (not diagnosed at enrolment)	Enrolled: *n* = 40 Data analyzed: 40
Browning et al., 2020 [42] Prospective cohort study (2 gr.)	Medical ICU (mechanically ventilated patients)	MLG: 64 (±12.96) CG:71 (±4.51)	Delirium risk (not diagnosed at enrolment)	Enrolled: *n* = 6 Data analyzed: 6
Correa et al., 2020 [39] Quasi-experimental study (2 gr.)	LTC institutions (patients with dementia/probable dementia)	IGPM: 85.1 (±8.7) CGCM: 85.3 (±7.6)	Probable dementia; (delirium symptom of advancing dementia)	Enrolled: *n* = 33 Data analyzed: 33
McCaffrey and Locsin, 2004 [37] RCT (2 gr.)	Postoperative orthopedic unit (hip/knee patients)	Total: 73.3 (±4.8)	Delirium risk (not diagnosed at enrolment)	Enrolled: *n* = 66 Data analyzed: 66
Cheong et al., 2016 [40] One-sample, within-subject	ACU (patients with delirium and dementia)	Total: 86.5 (±5.7)	Dementia with or without delirium	Enrolled: *n* = 25 Data analyzed: 25 (8 had delirium)
Sharda et al., 2019 [38] Pre-experimental (2 static gr.)	POSH clinic (postsurgical inpatients)	POSH: 75.0 CALM:74.6 (SD not reported)	Delirium risk (not diagnosed at enrolment)	Enrolled: *n* = 109 Data analyzed: 45
Helmes and Wiancko, 2006 [41] One-sample, within-subject (multiple case study)	ACU (geriatric assessment ward and family medicine ward patients)	Total: 82.7 (±7.4)	Diagnosis of dementia and delirium	Enrolled: *n* = 9, (2 had delirium) Data analyzed: 7 (including 2 with delirium)

**Abbreviations**: RCT: Randomized Controlled Trial; ICU: Intensive Care Unit; LTC: Long-Term Care; TICU: Trauma Intensive Care Unit; TOU: Trauma Orthopedic Unit; ACU: Acute Care Units; M-AMT: Memantine and Active Music Therapy group; M: Memantine group EG: experimental group; CG: control group; IMT: Interactive Music Therapy; PML: passive music listening; MLG: music listening group; IGMP: Intervention Group Popular Music; CGCM: Control Group Classical Music; POSH: Perioperative Optimization of Senior Heath group; CALM: Confusion Avoidance Led by Music group; *n*: number of participants; **Notes:** ^1^ The studies in this and all the other tables are listed according to their PEDro score—from the highest to the lowest quality. ^2^ Mean age and standard deviation (SD) values are reported according to the values available in the original included studies. Some studies reported the mean age/SD of the participants in each group, whereas others only reported the mean/SD age of all the participants. ^3^ The abbreviated names of the groups are presented in their original form, as identified in the articles.

**Table 2:** Interventions and delivery.

Study ^1^	Intervention ^2,3^ and Control	Primary Aims (Delirium-Related)	Dose and Delivery
Khan et al., 2020 [31]	EC: Personalized music listening (*n* = 17) EC: Pre-selected slow tempo music listening (*n* = 17) CC: Audiobook/attention control (*n* = 18)	Prevention and treatment (To impact the incidence and severity of delirium in ICU patients)	2 × 60 min p/day; 7 days (the same dose for all interventions)
Giovagnoli et al., 2018 [32]	EC: Active music therapy and Memantine (AMT) (*n* = 23) CC: Memantine (M) added to AchEI (*n* = 22)	Treatment (To affect language, global cognitive functioning, psycho-behavioral and social aspects, and daily activities of LTC patients)	AMT: 2 × 40 min p/week; 24 weeks. M: 20 mg per day
McCaffrey and Locsin, 2006 [36]	EC: Pre-selected music listening (*n* = 62) CC: Usual care (*n* = 62)	Prevention and treatment (To affect pain, cognition/acute confusion, the ability to ambulate and general satisfaction in postsurgical hip/knee patients)	Min. 1–4 × p/day, unreported duration; from awakening from anesthesia until discharge
McCaffrey 2009 [35]	EC: Pre-selected music listening (*n* = 11) CC: Usual care (*n* = 11)	Prevention and treatment (To affect cognitive function and acute confusion in postsurgical hip/knee patients)	Min. 4 × 60 min p/day; from awakening from anesthesia until discharge
Kim et al., 2020 [33]	EC: Interactive music therapy (IMT) (*n* = 44) EC: Passive, pre-selected, music listening (PML) (*n* = 44) CC: Usual care (*n* = 45)	Prevention (To prevent delirium through affecting sleep quality, melatonin/cortisol levels and pain in postsurgical ICU patients)	IMT: daytime (15–20 min), night-time (30 min). PML: night-time (30 min); from awakening until discharge
Johnson et al., 2018 [34]	EC: Pre-selected music listening (*n* = 20) CC: Usual care (*n* = 20)	Prevention and treatment (To affect delirium through decreasing physiologic variables in postsurgical patients)	2 × 60 min, p/day; 3 days (at 2 p.m. and 8 p.m.)
Browning et al., 2020 [42]	EC: Personalized music listening (*n* = 3) CC: Usual care (*n* = 3)	Prevention and treatment (To impact incidence and severity of delirium in ICU patients)	2 × 60 min p/day; 2 weeks
Correa et al., 2020 [39]	CC: Pre-selected Classical Music listening (*n* = 14) EC: Popular, Brazilian, personalized music listening (*n* = 19)	Treatment (To affect physiological, behavioral, and expressive outcomes in LTC patients with dementia/delirium)	4 × 20 min p/week (same dose for both interventions)
McCaffrey and Locsin, 2004 [37]	EC: Pre-selected music listening (*n* = 33) CC: Usual care (*n* = 33)	Prevention and treatment (To reduce delirium episodes in postsurgical hip/knee patients)	Max. 3 × 60 min p/day, (or at any time desired); from awakening until discharge
Cheong et al., 2016 [40]	EC: Creative Music Therapy (CMT) CC: The usual care (*n* = 25; 8 had delirium)	Treatment (To impact mood and engagement in AC patients with delirium/dementia)	CMT: 1 × 30 min p/day; 2 days
Sharda et al., 2019 [38]	EC: Confusion Avoidance Led by personalized Music (CALM) (*n* = 45) CC: Usual care (157)	Prevention and treatment (By affecting pain and anxiety to prevent/treat delirium in postsurgical inpatients)	CALM: Min. 2 × 20 min p/day, or at any time desired
Helmes and Wiancko, 2006 [41]	EC: Pre-selected music listening (Baroque music) CC: No music (2 trials of each condition compared in *n* = 9 participants; 2 had delirium)	Treatment (To reduce the frequency of disruptive behaviors in AC patients)	Minimum 4 × 30 min per day—minimum 3 days

**Abbreviations**: EC: experimental condition; CC: control condition; NR: not reported; ICU: Intensive Care Unit; LTC: Long-Term Care; TICU: Trauma Intensive Care Unit; TOU: trauma orthopedic; AC: acute care; M: Memantine; AchEI: acetylcholinesterase inhibitors/the usual pharmacological treatment; ATM: Active Music Therapy; ML: Music Listening; ITM: Interactive Music Therapy; PML: passive music listening; CMT: Creative Music Therapy; CALM: Confusion Avoidance Lead by Music; **Notes**: ^1^ The studies in this and all other tables are listed according to their PEDro score—from the highest to the lowest quality. ^2^ A more detailed description of the music interventions and the delivery procedures is given in the Supplementary Table S2. ^3^ The groups are defined and presented as experimental and control conditions (EC, and CC) with their particular content.

**Table 3:** Outcomes and assessment tools.

Study ^1^	Delirium Outcomes and Tools	Other Outcomes and Tools ^2^
Khan et al., 2020 [31]	OUTCOMES: Number of delirium-free/coma-free days and severity TOOLS: RASS; CAM-ICU; CAM-ICU-7	Anxiety (Face Anxiety Scale—VAS) Pain (CPOT) Physiological stress (HR, BP, RR) Sleep (STOP-BANG-RCS-Q) Mobility (physical/occupational therapy notes)
Giovagnoli et al., 2018 [32]	OUTCOMES: NR but delirium measured as one of the neuropsychiatric symptoms of advancing dementia TOOLS: NPI-Q	Language (SIB-L) Social interactions, memory, orientation, attention, praxis, visual–spatial ability and orientation (SIB) Independence in daily activities, instrumental activities (ADL and IADL) Psychic and behavioral symptoms of dementia (NPI-Q) Neurocognitive functions (MMSE) Perceived social support (LSNS)
McCaffrey and Locsin, 2006 [36]	OUTCOMES: Number of episodes of delirium/acute confusion TOOLS: Nurses’ narrative notes in medical records	Pain (numeric rating scale; number of pain medications) Ambulation (medical records and notes from nurses and physical therapists) Patient satisfaction (self-rating-post-discharge phone call.)
McCaffrey 2009 [35]	OUTCOMES: Presence and severity of delirium/acute confusion TOOLS: NEECHAM	Cognitive function (MMSE) Physiological measurements (oxygen saturation, BP, RR)
Kim et al., 2020 [33]	OUTCOMES: Incidence of delirium TOOLS: CAM-ICU	Quality and the duration of sleep (RCS-Q) Pain (NRS) Recovery after anesthesia (QoR-40) Cortisol and melatonin levels (Salivette tube)
Johnson et al., 2018 [34]	OUTCOMES: Presence of delirium/acute confusion TOOLS: CAM-ICU	Physiological measurements (SBP, HR, RR)
Browning et al., 2020 [42]	OUTCOMES: Incidence and severity of delirium TOOLS: CAM-ICU; RASS	NR
Correa et al., 2020 [39]	OUTCOMES: NR, but delirium measured as one of the neuropsychiatric symptoms of advancing dementia TOOLS: NPI-Q	Severity of neuropsychiatric manifestation (NPI-Q). Cardiovascular biofeedback (Cardio emotion) Facial expressions (FACS) Body movements (reactions grouped into body parts)
McCaffrey and Locsin, 2004 [37]	OUTCOMES: Number of delirium episodes TOOLS: Nurses’ notes and checklists	Ambulation (physiotherapists’ notes)
Cheong et al., 2016 [40]	OUTCOMES: NR, but delirium is assessed at baseline TOOLS: CAM	Engagement regulation (MPES) Mood regulation (OERS)
Sharda et al., 2019 [38]	OUTCOME: Incidence of delirium TOOL: ICD codes	Length of hospital stay (hospital records) Pain and mood (patient survey)
Helmes and Wiancko, 2006 [41]	OUTCOMES: NR, but delirium is assessed at baseline TOOLS: NR	Frequency and incidence of repetitive vocalizations/shouting and banging objects (systematic observations)

**Abbreviations**: NR: not reported; LAR: legally authorized representatives; NEECHAM: Neelon, Champagne, Carlson and Funk, (1996) acute confusion scale; RASS: Richmond Agitation and Sedation Scale; CAM: Confusion Assessment Method; CAM-ICU: Confusion Assessment Method for Intensive Care Units; CAM-ICU-7: Delirium Severity Scale; ICD: international classification of diseases; VAS: Face Anxiety Scale—Visual Analogue Scale; CPOT: Critical Care Pain Observation Tool; NPI-Q: Neuropsychiatric Inventory Questionnaire; SIB-L: Severe Impairment Battery Language; SIB: Severe Impairment Battery; ADL: Activities of Daily Living; IADL: Instrumental Activities of Daily Living; MMSE: Mini-Mental State Evaluation scale; LSNS: Lubben Social Network Scale; RCS-Q: Richard-Campbell Sleep Questionnaire; QoR-40: self-rating—The Quality of Recovery—40 questionnaire; Cardio emotion: Cardiovascular biofeedback; SBP: systolic blood pressure; HR: heart rate; RR: respiratory rate; FACS: Facial Action Coding System; MPES: Menorah Park Engagement Scale; OERS: Observed Emotion Rating Scale; NRS: numeric rating scale. **Notes**: ^1^ The studies in this and all other tables are listed according to their PEDro score—from the highest to the lowest quality. ^2^ Details of the assessment procedures for each outcome are given in the Supplementary Table S3.

## Data Availability

Most data generated or analyzed during this study are included in this published article and its supplementary information files. Any additional datasets supporting the findings of this study are available from the corresponding author on reasonable request.

## Supplementary Materials

The following supporting information can be downloaded at: https://www.mdpi.com/article/10.3390/brainsci12050568/s1, Supplementary Figure S1: Music exposure and delirium incidence (fixed effects meta-analysis); Supplementary Figure S2: Publication bias assessment; Supplementary Figure S3: Influence analysis; Supplementary Method S1: Full search strategy; Supplementary Method S2: Eligibility criteria; Supplementary Method S3: Data extraction categories; Supplementary Table S1: Risk of bias assessment and PEDro-scale criteria; Supplementary Table S2: Music intervention description and delivery procedures; Supplementary Table S3: Assessment procedures.
