# Supplementary material for: Music Interventions and Delirium in Adults: A Systematic Literature Review and Meta-Analysis
Source: Brain Sci. 2022 Apr 28;12(5):568. doi: 10.3390/brainsci12050568 (PMC9138821; doi:10.3390/brainsci12050568)
Supplement: Supplementary file 1 [file brainsci-12-00568-s001.zip › brainsci-1678584-supplementary.pdf]

Supplementary figures

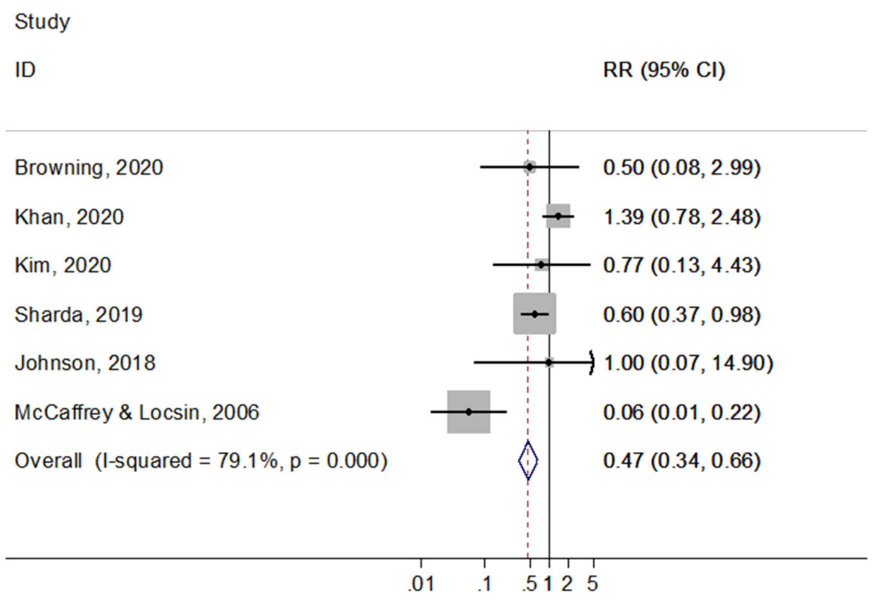

Supplementary figure S1. Music exposure and delirium incidence (fixed effects meta-analysis).

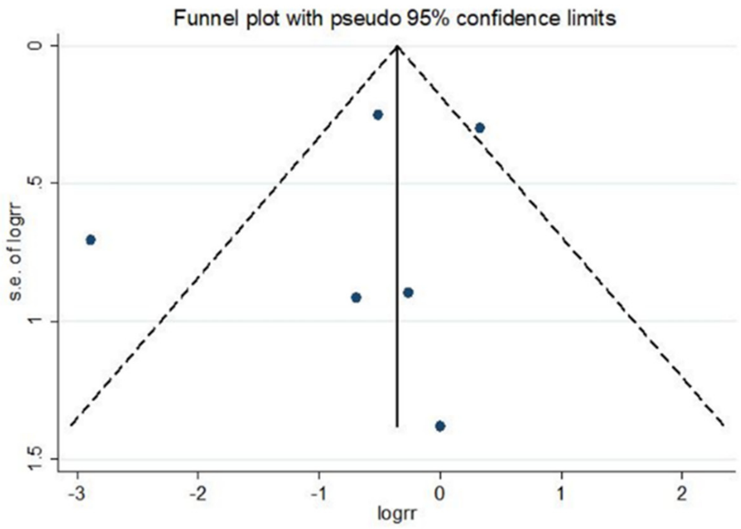

Egger's test, p-value=0.51

Supplementary figure S2. Publication bias assessment.

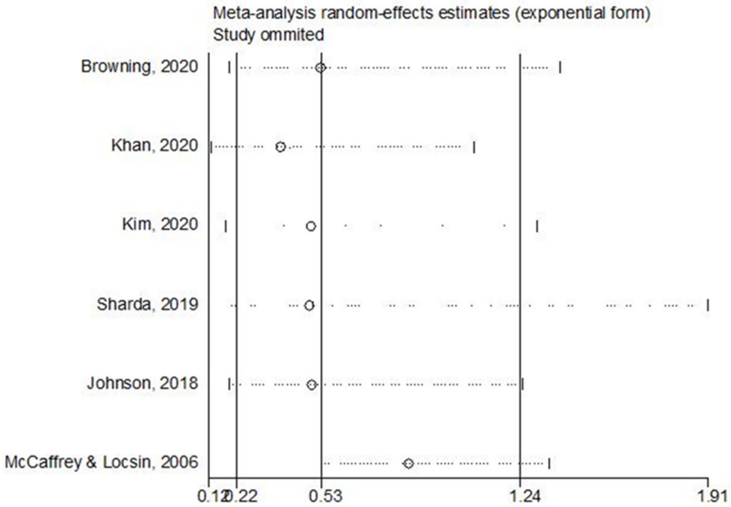

Study omitted	e^coef.	[95% Conf. Interval]	
Browning, 2020	0.52411324	0.19874562	1.3821422
Khan, 2020	0.38084498	0.13465166	1.0771713
Kim, 2020	0.4902105	0.18477046	1.3005668
Sharda, 2019	0.48443422	0.12256318	1.914739
Johnson, 2018	0.4943558	0.19554184	1.2497972
McCaffrey & Locsin, 2006	0.84168667	0.5271588	1.3438768
Combined	0.52544621	0.22232503	1.2418472

Supplementary figure S3. Influence analysis.

Supplementary methods

Supplementary method S1. Full search strategy

Database: Ovid MEDLINE(R) ALL (1946 – to present)

1 exp Delirium/

2 Alcohol Withdrawal Delirium/

3 Confusion/

4 delir*.mp.

5 confus*.ti.

6 (acute confusional state* or toxic confus* or altered mental status or acute psychosis or acute psychotic or icu psychosis or (intensive care unit* and psychosis) or clouded state or "clouding of consciousness" or toxic confus* or exogenous psycho* or toxic psycho* or acute encephalopathy or acute brain failure or acute organic psychosyndrome).mp.

7 (exp neurocognitive disorders/ or (cognitive disorder* or cognitive impairment* or cog-nitive dysfunction* or cognitive

failure*).ti.) and (exp Health Facilities/ or Inpatients/ or hospital*.hw. or (inpatient* or hospital*).ti.)

8 or/1-7

9 Music/

10 Music Therapy/

11 Singing/

12 Acoustic Stimulation/

13 Evoked Potentials, Auditory/

14 (music* or song* or sing or sings or singing* or singer* or chant* or melod* or acoustic stimulation* or auditory stimulation* or rhythmic vocalization* or piano or guitar* or vio-lin*).mp.

15 (vocal* or sound* or auditory or whistl* or rhythm*).ti.

16 or/9-15

17 8 and 16

Comments:

/ = MeSH (Medical Subject Heading)

.mp = multi-purpose, i.e searches several fields at once: title, abstract, subject heading etc.

.ti = title field

.hw = subject heading word, allows you to retrieve every Ovid Descriptor that includes a particular word.

Exp = explode. The MeSH term "Delirium" are "exploded", i.e. it retrieves automatically citations that carry the specified MeSH heading as well as the more specific term indented beneath it in the MeSH hierarchy: Emergence Delirium

APA PsycInfo

1 Delirium/

2 Delirium Tremens/

3 Mental Confusion/

4 delir*.mp.

5 confus*.ti.

6 (acute confusional state or toxic confus* or altered mental status or acute psychosis or acute psychotic or icu psychosis or (intensive care unit* and psychosis) or clouded state or "clouding of consciousness" or toxic confus* or exogenous psycho* or toxic psycho* or acute encephalopathy or acute brain failure or acute organic psychosyndrome).mp.

7 or/1-6

8 exp Music/

9 Music Therapy/

10 Singing/

11 Auditory Stimulation/

12 exp Music Perception/ or Musical Ability/

13 (music* or song* or sing or sings or singing* or singer* or chant* or melod* or acoustic stimulation* or auditory stimulation* or rhythmic vocalization* or piano or guitar* or vio-lin*).mp.

14 (vocal* or sound* or auditory or whistl* or rhythm*).ti.

15 or/8-14

16 7 and 15

Scopus

Advanced > Enter query string

(TITLE-ABS-KEY (delir*) OR TITLE (confus*) OR TITLE-ABS-KEY (“acute confusional state*” OR “toxic confus*” OR “altered mental status” OR “acute psychosis” OR “acute psychotic” OR “icu psychosis” OR (“intensive care unit*” AND psychosis) OR “clouded state” OR "clouding of consciousness" OR “toxic confus*” OR “exogenous psycho*” OR “toxic psycho*” OR “acute encephalopathy” OR “acute brain failure” OR “acute organic psychosyndrome”)) AND (TITLE-ABS-KEY (music* OR song* OR sing OR sings OR sing-ing* OR singer* OR chant* OR melod* OR “acoustic stimulation*” OR “auditory stimula-tion*” OR “rhythmic vocalization*” OR piano OR guitar* OR violin*) OR TITLE (vocal* OR sound* OR auditory OR whistl* OR rhythm*))

Supplementary method S2. Eligibility criteria

	INCLUSION	EXCLUSION
Participants	Adults (≥18) with or at risk of developing delirium, across medical settings and levels of care.	Younger adults (≤18)
Music intervention	Any type of music intervention (including listening to live or pre-recorded music, music making, singing, playing, improvising, music and movement, music and dance, relaxation to music, music therapy etc.).	Music is a component of an intervention, and the impact of music is not reported separately.

	Music interventions delivered and administered by either the medical staff, trained music therapists, musicians, or others.	The effects on the outcome measures for delirium cannot be clearly attributed to the music interventions.
Comparator	No limitations on the type of comparators, expected to find the studies in which the comparator is mainly "the usual care" or another intervention.	
Outcome measures	Incidence, severity and/or duration of delirium, any changes and improvements in general well-being related to delirium.<br>Delirium data is reported, regardless of whether the aim of the study was to investigate prevention or treating, and regardless of whether delirium was the main focus of the study.<br>Studies with mixed diagnoses where outcomes were reported separately for delirium;	Delirium or acute confusion not explicitly mentioned;
Methodolog y	Randomized controlled trials, controlled trials, and quasi-experimental studies, as well as observational studies;	Qualitative studies, program descriptions, surveys, systematic reviews or editorials
Publications	Full papers in peer-reviewed journal, those published as reports, higher degree theses and dissertations;	Ongoing studies, partially published research, studies that were informally reported and/or unpublished, book chapters and books where data was not reported.
Language	Studies in English, Norwegian, Swedish, Danish, Serbian (Croatian, Bosnian), Spanish and Italian.	

Supplementary method S3. Data extraction categories

DATE OF EXTRACTION
AUTHOR
TITLE
PUBLICATION TYPE
COUNTRY OF ORIGINE
SOURCE OF FINDING
LANGUAGE
AIM AND OBJECTIVES
STUDY DESIGN
INCLUSION/EXCLUSION CRITERIA
RECRUITMENT STRATEGY
UNIT OF ALLOCATION
THEORETICAL FRAMEWORK
AGE
GENDER
ETHNICITY
DISEASE
COMORBIDITIES
INTERVENTION(S) AND CONTROL
CLINICAL CONTEXT/LEVEL OG CARE
DOSE/FREQUENCY
DELIVERY SETTING
ADMINISTRATOR
PRIMARY OUTCOMES
SECONDARY OUTCOMES
MEASURING TOOLS
DATA COLLECTION AND MEASURING PROTOCOLS
PARTICIPANTS ENROLLED
INCLUDED IN ANALYSES
WITHDRAWALS/EXCLUSIONS
RESULTS OF ANALYSES
DEMOGRAPHIC data
COSTS
RESOURCES
ADVERSE EVENTS
SUITABILITY OF THE MEASURING TOOL
OTHER
Limitations?

Supplementary tables

Supplementary table S1. Risk of bias assessment and PEDro-scale criteria

	PEDro item number [a] ✓✗										
**Reference**	**1**	**2**	**3**	**4**	**5**	**6**	**7**	**8**	**9**	**10**	11	Total (/12)
Khan et al., 2020	✓	✓	✓	✓	✗	✓	✓	✓	✓	✓	✓	9
Giovagnoli et al. 2018	✓	✓	✓	✓	✗	✗	✓	✓	✓	✓	✓	8
McCaffrey & Locsin 2006	✓	✓	✓	✓	✗	✗	✗	✓	✓	✓	✓	7
McCaffrey 2009	✗	✓	✓	✓	✗	✗	✗	✓	✓	✓	✓	7
Kim et al., 2020	✓	✓	✗	✓	✗	✗	✗	✓	✓	✓	✓	6
Johnson et al., 2018	✓	✓	✗	✓	✗	✗	✗	✗	✓	✓	✓	5
Browning et al., 2020	✓	✓	✗	✗	✗	✗	✗	✓	✓	✗	✓	4
Correa et al., 2020	✓	✗	✗	✗	✗	✗	✗	✓	✓	✓	✓	4
McCaffrey & Locsin, 2004	✓	✓	✓	✗	✗	✗	✗	✗	✗	✓	✗	3
Cheong et al., 2016	✓	✗	✗	✗	✗	✗	✗	✓	✓	✗	✓	3
Sharda et al. 2019	✓	✗	✗	✗	✗	✗	✗	✗	✓	✓	✗	2

Helmet & Wiancko 2006	✗	✗	✗	✗	✗	✗	✗	✗	✓	✗	✗	1

[a]PEDro items: 1. eligibility criteria were specified. 2. subjects were randomly allocated to groups (in a crossover study, subjects were randomly allocated an order in which treatments were received). 3. allocation was concealed: 4. the groups were similar at baseline regarding the most important prognostic indicators. 5. there was blinding of all subjects. 6. there was blinding of all therapists who administered the therapy. 7. there was blinding of all assessors who measured at least one key outcome. 8. measures of at least one key outcome were ob-tained from more than 85% of the subjects initially allocated to groups. 9. all subjects for whom outcome measures were available received the treatment or control condition as allo-cated or, where this was not the case, data for at least one key outcome was analysed by "in-tention to treat". 10. the results of between-group statistical comparisons are reported for at least one key outcome. 11. the study provides both point measures and measures of variabil-ity for at. least one key outcome. NB. Item 1 not included in total score.

Supplementary table S2. Music intervention description and delivery procedures

STUDY[1]	MUSIC INTERVENTION DESCRIPTIONS AND DELIVERY PROCEDURES
Khan et al., 2020	• Personalized music (PM) was assessed from the legally authorized representatives (LAR), pre-intervention, using Music-preference Assessment Tool (MAT).<br>• Non-personalized, relaxing slow tempo music (STM) (60-80bpm) consisted of guitar, piano, classical music, Native American flute sounds, pre-selected by a board-certified music therapist (MT).<br>• Attention control (audiobook).<br>All the interventions were delivered through noise-cancelling headphone and MP3 player devices.
Giovagnoli et.al., 2018	• Active music therapy (AMT) was given in addition to Memantine (M) drug, and involved a non-verbal approach and free sound-music interactions, using rhythmical and melodic instruments (xylophones, glockenspiels, triangles, wind-chimes, maracas, small woods, guiros, and ethnic percussions). Each session began with musical improvisation inviting patients to choose an instrument and to play using a free technique.
McCaffrey & Locsin, 2006	• The first music listening (ML) CD placed on the player was a lullaby musical selection while the patients were awakening from the anaesthesia.<br>• After they were awake the patients could choose from any of the CDs from the preselection provided by the researchers. It isn't reported how the music selection was made.<br>Interventions were delivered via CD player placed by the bedside.
McCaffrey, 2009	• A music listening (ML) CD containing "soothing lullaby music" was delivered immediately upon the arrival at the orthopaedic floor from the recovery area, and played continuously from a CD player.<br>• As soon as they were awake the patients could choose from a variety of music provided by the researchers (a variety of genres, styles, artists, etc.). It isn't reported how the music selection was made.<br>Interventions were delivered via CD player placed by the bedside.
Kim et al., 2020	IMT intervention consisted of individual music therapy during the day (15-20min) and personalized music-listening (PML) (30min), following a music therapist's assessment of willingness to listen and preferences, at night.<br>• PML intervention was delivered only night-time. Music consisted of several pre-selected relaxing classical pieces previously utilized for their relaxing properties in other studies, that patients could choose from.<br>All music listening was facilitated through earphones and MP3player.
Johnson et al., 2018	• Pre-recorded, researcher-selected music, with slow tempo, low pitch and simple repetitive rhythms was played for the participants through an IPod and headsets, over 3 days upon admission. The intervention was standardized.

Browning et al., 2020	• Therapeutic music listening (ML) delivered as a nursing intervention, involving patient-specific, passive listening to pre-recorded music. The music listening content was selected in collaboration between the patients, family, and PI.
Correa, et al., 2020	• The tracks for the popular/familiar music group (IGPM) were based on the assessed music preferences of the participants. The intervention was delivered in a silent room, previously prepared, and accompanied by the notebook of songs previously selected for each participant.<br>• Classical piano music was selected for the other group (CGCM).<br>Both interventions were delivered through a Sony Headphone of the over-ear type and the volume in the headphones had a frequency of 60-70 decibels (corresponding to the volume of normal conversation).
McCaffrey & Locsin, 2004	• Researcher-selected pre-recorded music for patients to choose from.<br>The intervention was delivered through a bed-side CD player placed within patients' reach, that could be automatically turned on.
Cheong, et al., 2016	• The CMT intervention consisted of active, music improvisation such as spontaneous music making, and playing familiar songs of patient's choice.
Sharda, et al., 2019	• An iPod shuffle with personalized music lists was prepared and delivered along with the headphones as a part of CALM intervention.<br>Intervention was delivered either through disposable earbuds, disposable over the ear headphones, or reusable over the ear headphones.
Helmes & Wiancko, 2006	• Baroque music was pre-selected by the researchers because of its rhythmic nature and absence of sharp transitions in volume. It consisted of orchestral pieces by Albinoni, Pachelbel, and Bach.<br>• A minimum of 2 trials of each condition was delivered to each participant in randomized order.<br>Music was played in the room from a portable compact disc player.

Abbreviations: PM: Personalized music; STM: Slow Tempo Music; MAT: Music Assess-ment Tool[49]; LAR: Legally authorized representatives; MT: Music Therapist; AMT: Ac-tive Music Therapy; M: Memantine drug added to AchEI – acetylcholinesterase inhibi-tors/the usual pharmacological treatment; ML: Music Listening; IMT: Interactive Music Therapy; PML: Passive music listening; PI: Primary Investigator; IGMP: Intervention Group Popular Music; CGCM: Control Group Classical Music; CMT: Creative Music Therapy; CALM: Confusion Avoidance Led by Music. **Notes**: [1] The studies in this and all other tables are listed according to their PEDro score – from the highest to the lowest quality.

Supplementary table S3. Assessment procedures

STUDY[1]	ASSESSMENTS PROCEDURES FOR EACH OUTCOME
Khan et al., 2020	• Delirium/delirium severity: assessed at enrolment; twice daily until discharge or day 28 - after audio interventions); and 72h after mechanical ventilation (RASS, CAM-ICU, CAM-ICU-7)).<br>• Anxiety: assessed once daily (after morning intervention) using self-report Visual Analogue Scale, (VAS-Face Anxiety Scale).<br>• Pain: assessed twice daily (after each intervention) using CPOT.<br>• Vital signs: HR, BP, RR recorded before and after each session.<br>• Sleep: patients screened for sleeping apnoea (STOP-BANG, Richards-Campbell Sleep Questionnaires) during intervention and 72h after mechanical ventilation.<br>• Mobility: assessed from the inpatient occupational/physical therapy notes.<br>• Critical care recovery centre follow-up: 90 days after discharge.
Giovagnoli et.al., 2018	• Delirium: measured as one of the dementia features in NPI-Q at baseline and at 12 and 24 weeks.<br>• Other outcomes: also assessed at baseline, and at weeks 12 and 24 (the patients were evaluated blindly by a neuropsychologist).
McCaffrey & Locsin, 2006	• Acute confusion: assessed from the nurses' narrative postoperative notes.

	• Postoperative pain: numerical rating on a scale 1-10 by nurses every 8 hours; the number of pain medications received by each patient after the discontinuance of the patient-controlled analgesia pump on the first postoperative day.<br>• Readiness to ambulate: assessed by the physical therapist (right after the surgery). The score was based on the patient's cognitive status, pain and willingness to participate in his/her own recovery. The distance ambulated on each postoperative day assessed from physical therapy notes.<br>• Patient-satisfaction: assessed during a post-discharge phone-call (2weeks later); patients were asked to rate their hospital experience on a scale 1-10.
McCaffrey, 2009	• Acute confusion: assessed preoperatively as well as during the first 3 postoperative days.<br>• Cognitive function: assessed with MMSE preoperatively, and on the 3 consecutive postoperative days.<br>• Physiological measurements: obtained by measuring physiological factors (oxygen saturation, blood pressure, pulse, and respiration).
Kim et al., 2020	• Delirium: Subjects were screened for postoperative delirium three times a day during ICU stay.<br>• Saliva melatonin and cortisone levels: measured 3 times, on preoperative, operation day, and postoperative day1 (POD1).<br>• Sleep quality: RCSQ and QoR-40 assessments conducted on the preoperative day and POD1 and 2.
Johnson et al., 2018	• Delirium: screened on admission and every 12 hours at the beginning of each shift.<br>• Physiological measurements: collected on admission and every four hours over a three-day period.
Browning et al., 2020	• Delirium: RASS and CAM-ICU assessments were performed by the PI during the prescribed dosing intervals, and recorded in the patients' charts every 8 to 12 hours (or as needed with any change in clinical status).
Correa, et al., 2020	• Neuropsychiatric manifestations: the NPI questionnaire administered to family members and/or care givers for pre/post-intervention evaluation.<br>• Body movements/facial expressions: systematic observations/recordings during interventions, individually, weekly.<br>• Cardiovascular biofeedback: recording the time intervals between heartbeats through an external sensor placed on the fingers or the auricular lobe, the heart rate and the frequency at which the participants maintained emotional balance (cardiac coherence) was also assessed (before and after intervention).
McCaffrey & Locsin, 2004	• Delirium: assessed from the containing the nurses' notes on episodes of confusion, disorganized thinking, altered level of consciousness or cognitive disturbances.<br>• Readiness to ambulate: assessed from physiotherapists' notes (on the day of surgery). One of the measures for ambulation was also that the patient is alert and oriented to time, place and person.
Cheong, et al., 2016	• Mood & Engagement: patients assessed for 3 consecutive days (day 1=baseline, days 2&3 = intervention days). DAY 1- 90, during the usual care; DAYS 2 & 3 - 30 min before, 30 min during and 30 min after the intervention.<br>• The MPES and OERS were rated for each patient with 5-min intervals (1 point accorded for the most frequently observed behaviour in each scale during 5 min interval).<br>• Two raters coded affect and mood independently but simultaneously, so that both affect and mood data could be available for the same timeframe.
Sharda, et al., 2019	The two groups were compared at baseline and post-intervention.<br>• Baseline: demographics, cognitive status, depression, hearing deficits, Laparoscopic procedure.<br>• Post- intervention: delirium incidence, discharge disposition and length of stay, patient-survey.
Helmes & Wiancko, 2006	• Frequency and incidence of disruptive behaviours: the number of bangs, shouts, or uses of the call bell per minute observed/recorded during the intervention trials and non-intervention trials, at random hours (between 10 a.m. and 5 p.m.);<br>• Each participants observed for a minimum of 4 trials a 30-min periods, on a minimum of 3 successive days.

Abbreviations: NEECHAM: Neelon, Champagne, Carlson & Funk, (1996) acute confusion scale; RASS: Richmond Agitation and Sedation Scale; CAM: Confusion Assessment Meth-od; CAM-ICU: Confusion Assessment Method for Intensive Care Units; CAM-ICU-7: Delir-ium Severity Scale; ICD: International classification of diseases; VAS: Face Anxiety Scale- Visual Analogue Scale; CPOT: Critical Care Pain Observation Tool; NPI-Q: Neuropsychiat-ric Inventory Questionnaire; SIB-L: Severe Impairment Battery Language; SIB: Severe Im-pairment Battery; ADL: Activities of Daily Living; IADL: Instrumental Activities of Daily Living; MMSE: Mini-mental State Evaluation scale; LSNS: Lubben Social Network Scale; RCS-Q: Richard-Campbell Sleep Questionnaire; QoR-40: self-rating-The Quality of Recov-ery - 40 questionnaire; Cardio emotion: Cardiovascular biofeedback; SBP: systolic blood pressure; HR: heart rate; RR: respiratory rate; FACS: Facial Action Coding System; MPES: Menorah Park Engagement Scale; OERS: Observed Emotion Rating Scale; NRS: numeric rating scale. **Notes**: [1] The studies in this and all other tables are listed according to their PED-ro score – from the highest to the lowest quality.
